# Osteoporosis: Modern Paradigms for Last Century’s Bones †

**DOI:** 10.3390/nu8060376

**Published:** 2016-06-17

**Authors:** Marlena C. Kruger, Frances M. Wolber

**Affiliations:** School of Food and Nutrition, Massey Institute of Food Science and Technology, Massey University, Palmerston North 4442, New Zealand

**Keywords:** osteoporosis, bone health, ageing, bone mineral density

## Abstract

The skeleton is a metabolically active organ undergoing continuously remodelling. With ageing and menopause the balance shifts to increased resorption, leading to a reduction in bone mineral density and disruption of bone microarchitecture. Bone mass accretion and bone metabolism are influenced by systemic hormones as well as genetic and lifestyle factors. The classic paradigm has described osteoporosis as being a “brittle bone” disease that occurs in post-menopausal, thin, Caucasian women with low calcium intakes and/or vitamin D insufficiency. However, a study of black women in Africa demonstrated that higher proportions of body fat did not protect bone health. Isoflavone interventions in Asian postmenopausal women have produced inconsistent bone health benefits, due in part to population heterogeneity in enteric bacterial metabolism of daidzein. A comparison of women and men in several Asian countries identified significant differences between countries in the rate of bone health decline, and a high incidence rate of osteoporosis in both sexes. These studies have revealed significant differences in genetic phenotypes, debunking long-held beliefs and leading to new paradigms in study design. Current studies are now being specifically designed to assess genotype differences between Caucasian, Asian, African, and other phenotypes, and exploring alternative methodology to measure bone architecture.

## 1. Introduction

Bone tissue undergoes continuous change throughout life via a series of processes. Bone is destroyed and resorbed by osteoclasts, then replaced through the formation of new bone by osteoblasts. Mineralisation is carried out by osteoblasts, while being directed by the osteocytes and osteoclast activity [1]. Bone turnover and bone remodelling are both tightly linked and tightly regulated through bone forming and resorptive activities by osteoblasts and osteoclasts, respectively.

An imbalance between bone resorption and bone formation resulting in a decrease in bone mineralisation is termed osteopenia. This can further progress to osteoporosis and cause structural failure. Osteoporosis is a generalised skeletal disorder characterised by decreased bone mass and deteriorated bone architecture. Osteoporosis results in an increased susceptibility to bone fractures, and accelerated bone loss is correlated with an increased post-fracture mortality risk [2]; thus, osteoporosis is a major health concern.

The amount of bone present in the body, bone mineral content, and bone mineral density are parameters measured to determine whether a person is osteoporotic. Bone strength is dependent on both the quantity of minerals present (BMD) and the quality of the bone. Bone remodelling is a major determinant of bone strength. Bone quality is a function of bone morphology and architecture as well as of bone material properties. Clinically, the gold standard for measuring bone strength and bone quality is dual energy X-ray absorptiometry (DXA) [3,4]. DXA measures the volume of bone in the body and the amount of mineral in the bone, from which the bone mineral density (BMD) of the individual bone sections can be determined. The BMD measurement generates a T-score, for which a score of 0 is normal, a score of <−1.5–−2.5 is diagnostic of osteopenia, and a score of <−2.5 is diagnostic of osteoporosis [4]. It has been shown that over 50% of patients with fragility fractures have a T-score <−2.5 [5].

The incidence of osteoporotic fractures is rising globally at an alarming rate. In 2010, the annual mean incidence of non-traumatic fractures in people aged 50+ across North America, Europe, Australia, and Japan was found to be approximately 6700 per 100,000 people [6]—that is, 6.7% of older adults could be expected to experience a non-traumatic fracture in any given year. The true rates are likely to be higher, given it is estimated that up to one-third of vertebral fractures are missed on initial radiograph reading and thus go undiagnosed [7]. These fractures represent a heavy personal, social, and economic burden. In New Zealand alone, the cost of treatment and management of osteoporosis is projected to be over $450M annually by the year 2020 [8].

## 2. Review

The implementation of widespread clinical use of bone biomarkers over the last few decades has led to improved understanding of the epidemiology of osteoporosis. The classical paradigms of osteoporosis were that it was largely confined to Caucasian women who are elderly and slender, and that it was caused by the natural post-menopausal reduction in oestrogen reduction combined with calcium deficiency or an insufficient vitamin D status. However, more recent research has demonstrated that osteoporosis is not limited to a single race, sex, age, or body type. These are discussed individually below.

### 2.1. Ethnicity

It has become apparent that osteoporosis is not confined to Caucasians. The annual incidence of non-traumatic vertebral fractures in American women is no more than 1.7-fold that for Japanese women [6]. The disparity is even less evident in women aged 70+, with estimates of 3741 versus 4364 non-traumatic hip, vertebral, and other fractures per 100,000 annually in Japan and the USA, respectively.

**Figure 1:**
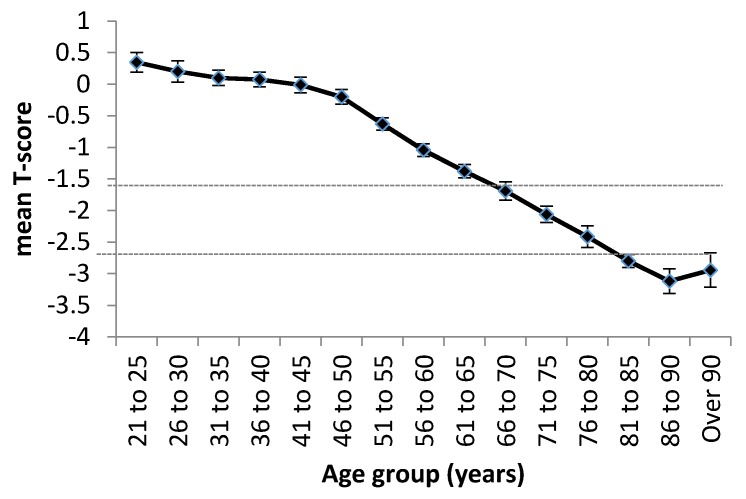
Mean T-scores by age group of a total of 598,757 women in Asia. Data are shown as mean ± SEM ofseven countries (Singapore, Taiwan, Thailand, Vietnam, Malaysia, Indonesia, and the Philippines). Dotted horizontal lines depict T-score cut-offs for osteopenia (−1.5) and osteoporosis (−2.5). Figure republished with permission [9].

A study of women across seven Asian countries (Singapore, Taiwan, Thailand, Vietnam, Malaysia, Indonesia, and the Philippines) using the Lunar Achilles heel scanner demonstrated that the mean T-score of the women assessed decreased dramatically with ageing (Figure 1). The authors noted that over 50% of women aged 55+ were osteopenic (T-score <−1.5) or osteoporotic (T-score <−2.5) [9]; by the age of 70, more than half were frankly osteoporotic.

It was also observed that the population incidence differed between countries, with women from the Philippines and Indonesia having the lowest T- scores. Vietnamese women experienced the fastest rate of decline in T-scores, suggesting that osteoporosis will be a major problem in this country in the near future. Likewise, osteoporosis is a growing problem in India, with an estimated prevalence of 20% in women over age 50; this equates to approximately 46 million Indian women [10].

Similarly, the incidence of fragility fractures in a major Japanese city has been shown to increase over the last three decades [11]. The incidence of limb fractures in this population was lower than in Caucasians in Northern Europe, but vertebral fractures were higher. Thus, osteoporosis is a global problem, and is certainly not isolated within a single race or genotype. It is also a complex problem and can manifest as spinal, hip, radius, and/or humeral fracture. Osteoporosis incidence is partly dependent on ethnicity, but quantifying osteoporosis risk is impacted further by emigration and whether or not immigrants retain the diet and culture of their home country. For example, forearm fracture risk was shown to be higher in ethnic Norwegians compared to immigrants from Asia to Norway [12]. In another study, the hip fracture rate in native-born Swedes was found to be nearly twice as high as those of immigrants to Sweden [13].

It remains unclear what are the relative contributions of genetic ethnicity versus cultural ethnicity to osteoporosis risk, adding to the complexity of assigning osteoporosis risk within a geographical or cultural region. However, this is an important aspect of osteoporosis to factor into data analysis, given the ever-increasing rate of global relocation and emigration by individuals, particularly in Europe. While health interventions are likely to be responsible at least in part for the very recent declines noted in hip fracture incidences in many countries, another contributor may be increasing numbers of immigrants with lower racial incidences of osteoporosis, and this could be a confounding factor when assessing the value of intervention strategies [14,15].

### 2.2. Sex

**Figure 2:**
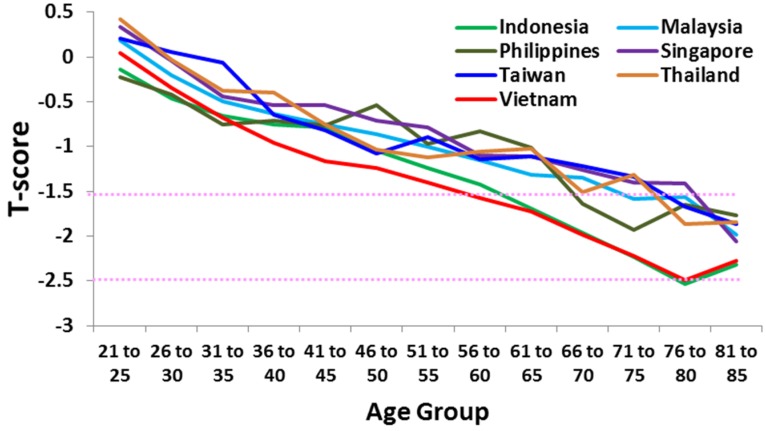
Mean T-scores by age group of men in Singapore (N = 6563), Taiwan (N = 6714), Thailand (N = 3150), Vietnam (N = 35,320), Malaysia (M = 59,458), Indonesia (N = 36,594), and the Philippines (N = 25,527) [16]. Dotted horizontal lines indicate osteopenia (−1.5) and osteoporosis (−2.5).

Likewise, osteoporosis is not confined to women. The Lunar Achilles study [9] found that more than 50% of Asian men over 45 years assessed were osteopenic or osteoporotic. Men from Indonesia and Vietnam had the lowest T-scores (Figure 2), again highlighting Vietnam as a country whose population is likely to experience a significant rise in osteoporosis in the near future.

**Figure 3:**
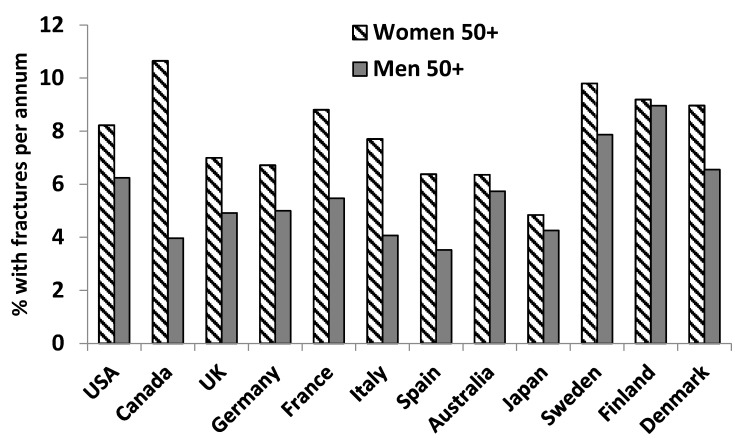
Non-traumatic fracture incidence (per 100 people) of hip, vertebral, and other bones for women versus men aged 50+ in select countries in 2010. Graph compiled from data published in Wade *et al*. [6].

Men in Europe, the Middle East, and Asia have been shown to be at high risk of hip fractures (defined as >150 fractures per 100,000 men) [17]. In a number of countries, non-traumatic fracture incidence in older men approaches that of women (Figure 3). In addition, men have been shown to have a higher mortality incidence after hip fracture than women [18,19].

### 2.3. Age

It remains true that osteoporosis is a disease that manifests in the elderly. The osteoporotic fracture incidence in women aged 50+ has been shown to approximately double with each additional decade of life [6] and, as the elderly population increases, so too will the incidence and prevalence of osteoporosis.

However, while bone loss occurs at an increasing rate with ageing, bone mass is acquired far earlier in life. Approximately one-third of adult mineral is deposited in the bone during adolescence. Bone density consolidation continues through young adulthood, with bone mass in the lumbar spine and femoral neck peaking at the end of the second decade of life and whole-body bone mass peaking in the third decade [20,21]. Low acquisition of mineral mass during this period is associated with an increased risk of osteoporotic fracture [22,23]. Childhood bone parameters at age 8–11 years are, however, a poor predictor of peak bone mass at age 18–19 [24], suggesting that modifiable factors during the earlier teen years play key roles in bone development. A number of lifestyle choices directly contribute to accretion of bone mass during childhood and adolescence; evidence is strongest for the positive influences of physical activity and calcium intake [25,26,27]. There is also good evidence for positive correlations between bone mass and vitamin D and dairy consumption, and a negative correlation between bone mass and DMPA contraceptive injections [20]. Eating disorders common in adolescents, such as bulimia nervosa and anorexia nervosa, significantly reduce bone density [28]. Osteoporosis that manifests in the elderly is seeded in childhood and, thus, this period of growth is the best time to target intervention strategies in order to increase bone mineral content and bone area in the general population.

### 2.4. Body Phenotype

The mechanical effect of adipose tissue on bone and oestrogen synthesis by adipocytes were long believed to be the main causal reasons for the lower incidence of osteoporotic fractures observed in obese or overweight women [29]. In contrast, underweight women were reported to be particularly susceptible to effects of health and lifestyle factors on bone, as evidenced by statements such as “The influence of smoking on the female skeleton seems mainly to be caused by the associated slenderness” [30] and “Type 2 diabetes and hypertension seem to be associated with increased bone density…in lean elderly women” [31].

However, body mass index (BMI) does not always correlate with bone health, particularly in diabetic women [32]. While bone turnover rate may be affected by body weight [33], body weight does not accurately predict fat mass or lean mass. Indeed, more rigorous analysis has now demonstrated that lean mass, rather than fat mass, is the main factor correlating with bone density. Increased adiposity in girls prior to puberty correlated with increased bone strength during adolescence, but the effect was lost when lean mass was taken into account [34]. As shown in Table 1, a study in urban black South African women demonstrated using multivariate regression that lean mass was a greater predictor than fat mass of BMD and fracture risk [35].

### 2.5. Oestrogen

The hormonal transition through menopause is a key risk factor for osteoporosis development. Surgically-induced menopause in young women has been shown to alter bone metabolism and favour osteoporotic onset with a concurrent increase in body mass index [36]. However, a recent study in Singaporean Chinese women that assessed osteoporosis in conjunction with physical exercise and body mass noted that “bone status after menopause may not be worse than that dictated by age alone” [37]. It is difficult to determine the relative contributions of age and oestrogen loss to osteoporosis as they occur concurrently and are impacted by other factors such as ethnicity, BMI, diet, and lifestyle.

The purported bone-protective effect of oestrogen-producing fat tissue, and the high incidence of osteoporosis in post-menopausal women, led to hormone replacement therapy (HRT) being recommended to prevent or halt the progression of osteoporosis in the 1980s [38]. The clinical use of HRT fell out of favour a few decades later due to safety concerns of HRT being associated with an increased risk of breast cancer recurrence [39,40]. More recent analysis has demonstrated that the randomized clinical trials on which these concerns were based were limited in scope and design, and resulted in conflicting evidence [41]. In addition, meta-analyses have shown no correlation between HRT and increased risk of endometrial cancer recurrence [42], ovarian cancer recurrence [43], or lung cancer [44]. However, HRT usage remained controversial and thus soybean protein, which contains phytoestrogens such as daidzein, was put forth as a possible alternative.

A strong correlation was shown between a lower incidence of osteoporosis and high intake of tofu and other soy products in both Japanese and Chinese postmenopausal women [45,46]. However, no effect of soy protein on bone resorption markers was found in Australian women [47]. This perplexing contradiction was explained by findings in later studies that approximately 50% of Asians and 75% of non-Asians lack the ability to absorb soy isoflavones and to metabolise daidzein to equol [48,49,50] due to a combination of the individual’s genotype, phenotype, and gut microbiota composition [51,52,53]. It has been suggested that modification of gut microbiota through diet could alter daidzein metabolism [54,55,56]. These findings demonstrate that genetic, as well as phenotypic, factors partially determine at the individual level both propensity for osteoporosis and response to oestrogenic preventative or treatment regimens.

### 2.6. Calcium

Calcium is a key component of bone, and calcium deficiency therefore is strongly associated with osteoporosis [57]. Many people fail to meet the recommended daily intake of calcium (800–1000 mg per day) due to inadequate diet, impaired absorption, or food intolerances [57,58]. Vitamin D_3_, whose active form 1,25(OH)_2_D_3_ is a cofactor required for calcium absorption in the gastrointestinal tract, is also strongly correlated with bone density [59]. Vitamin D insufficiency (25(OH)D_3_ serum levels <50 nmol/L) is also not uncommon due to diet, lack of sun exposure, or genetic mutations [60], and many studies have shown that the majority of postmenopausal women in areas around the world have insufficient vitamin D levels [61,62,63].

However, calcium supplementation has proven unsuccessful in preventing or halting the progression of osteoporosis. Ingestion of calcium citrate results in a bolus of calcium being absorbed rapidly, resulting in a sharp increase in blood calcium and giving rise to the term “calcium supplement syndrome” [64]. This exaggerated fluctuation in calcium homeostasis results in an increased risk of developing kidney stones and/or cardiovascular disease [65], but has no proven positive effect on bone formation [66,67]. Due to this, calcium supplements either for children or adults are no longer recommended by many health organisations worldwide [58,68,69].

It is now recognised that deficient dietary calcium intake does not always correlate with the level of bone loss [69]. This is likely to be due in part to the fact that the response to low calcium in the body, like soy isoflavone metabolism, differs widely between individuals. Calcium deficiency is classically countered in the body by the loop response, which involves increases in calcium absorption in the gut, calcium reabsorption in the kidney, and calcium withdrawal from the skeleton. However, ethnic differences in these responses have been observed [70,71]. This is likely to explain, at least in part, why, under circumstances of lower dietary calcium and vitamin D intake, African-Americans have a higher bone mineral density and develop osteoporosis at a lower frequency than their European-American counterparts [72]. Likewise, Chinese-American adolescents have been shown to absorb calcium at a higher level than non-Asian American adolescents [73].

**Figure 4:**
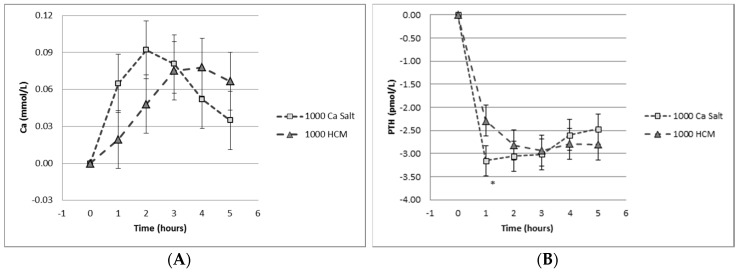
Effect of calcium delivery mode. Graphs depict the response 0–6 hours after the oral intake of 1000 mg calcium salt versus 1000 mg calcium in milk on (**A**) serum calcium and (**B**) serum parathyroid hormone (PTH). Reprinted with permission [74].

However, increasing dietary calcium, as opposed to calcium supplementation in tablet form in conjunction with adequate vitamin D supply, remains a key strategy for reducing osteoporosis. Dietary calcium in milk is absorbed differently to ionized calcium, so that even highly fortified calcium-containing milk remains safe for delivering calcium without perturbing calcium homeostasis, as shown in Figure 4 [74].

## 3. Discussion

Additional lifestyle factors that have recently been identified as affecting osteoporosis incidence are sleep quality and work shifts. Sleep duration was found to inversely correlate with bone mineral density in Korean women [75]. Nurses who worked night shifts or rotating shifts were shown to have a significantly higher incidence of wrist and hip fractures, lower bone mineral density, and a higher risk of osteopenia [76,77,78]. More recent research has identified melatonin, which regulates circadian rhythm, as being directly responsible for modulating bone mass; indeed, treatment with melatonin significantly increased BMD in osteopenic postmenopausal women [79]. More research in this area is warranted.

Recent studies have also identified food components that can impact bone resorption markers and bone health. Increased intake of specific herbs and green vegetables have reduced urinary calcium and significantly reduced the bone resorption marker CTx-1 in women with osteopenia [80]. Kiwifruit fed to rodents with ovariectomy-induced osteoporosis significantly reduced serum levels of CTx-1 and mRNA expression of receptor activator of nuclear factor kappa-B ligand (RANKL), an osteoclast differentiation factor that induces bone resorption [81,82].

Magnetic resonance imaging, high-resolution computed tomography (CT), and micro-CT are currently being evaluated as alternative or complementary strategies to DXA for measuring bone health [83]. Metabolomics are also being investigated to identify metabolite changes that may serve as early prognostic markers for osteoporosis [84]. Future strategies in the field of osteoporosis are likely to involve diet and lifestyle strategies targeted to specific ethnic and even individual genotypes. National and international strategies will include the development of supportive environments to improve childhood nutrition, with the goal of ensuring that peak bone mass is established during the growing years so as to reduce osteoporosis incidence in adulthood [85]. However, all studies and strategies will need to incorporate systems thinking, as the incidence and development of osteoporosis is not dependent on a single factor, but rather is due to the composite of race, sex, age, body type, diet, and lifestyle.

## 4. Conclusions

Osteoporosis is a disease that affects men as well as women. Although it manifests most commonly in the elderly, it often has its origins in childhood and young adulthood when the resulting peak bone mass is low. Thus local, national, and global strategies for prevention need to be directed at the adolescent and later teen years. Body fat does not protect against osteoporosis development, and oestrogen supplementation is not feasible for preventing or treating osteoporosis in males or pre-menopausal females.

Osteoporosis is a global health problem, but a single, global solution is not the answer due to the powerful effect of genetic differences on the development and manifestation of this disease. Instead, new approaches will be needed. Research is needed to identify diets, food components, sun exposure, sleep patterns, work shifts, and other modifiable factors that can impact one or more mechanisms within the complex, multifaceted pathophysiology of osteoporosis. Much work remains to be done in identifying differences in osteoporosis causes and incidences due to genotype and phenotype. It is likely that, in the future, screening for single nucleotide polymorphisms (SNPs) within the master genes driving key pathways of bone development may be used to identify at-risk patients [86]. Finally, any preventative or intervention strategies will be more effective with new and optimised ways to measure bone density and architecture, and new early-stage prognostic biomarkers of osteoporosis.

## Figures and Tables

**Table 1:** Association between bone mineral density (BMD) or fracture risk as the dependent variable, and body composition parameters as the independent variables, using two different models of multivariate regression. Table reproduced with permission [35].

	Fat Mass	Fat Mass	Fat Mass Adjusted	Lean Mass	Lean Mass	Lean Mass Adjusted
	β	*p*	*R*^2^	β	*p*	*R*^2^
Femoral Neck BMD						
Model 1	0.46	<0.001	0.20	0.51	<0.001	0.26
Model 2	0.39	<0.001	0.35	0.49	<0.001	0.40
Spine BMD						
Model 1	0.44	<0.001	0.19	0.51	<0.001	0.26
Model 2	0.38	<0.001	0.25	0.48	<0.001	0.30
Hip BMD						
Model 1	0.54	<0.001	0.29	0.58	<0.001	0.33
Model 2	0.50	<0.001	0.36	0.59	<0.001	0.40
Fracture risk						
Model 1	−0.24	0.001	0.05	−0.31	<0.001	0.09
Model 2	−0.16	0.05	0.15	−0.19	0.04	0.15
